# The Impact of Aortic Valvular Calcium on Transcatheter Heart Valve Distortion

**DOI:** 10.1155/2021/8829906

**Published:** 2021-01-05

**Authors:** Akihiro Nakajima, Toru Naganuma, Haruhito Yuki, Hirokazu Onishi, Tatsuya Amano, Hiroto Yabushita, Hiroyoshi Kawamoto, Satoru Mitomo, Yosuke Kitanaka, Tatsuya Nakao, Naoyuki Kurita, Hisaaki Ishiguro, Satoko Tahara, Masaaki Okutsu, Shotaro Nakamura, Sunao Nakamura

**Affiliations:** ^1^Interventional Cardiology Unit, New Tokyo Hospital, Chiba, Japan; ^2^Department of Cardiovascular Medicine, Graduate School of Medical Sciences, Kumamoto University, Kumamoto, Japan; ^3^Cardiovascular Surgery Unit, New Tokyo Hospital, Chiba, Japan

## Abstract

**Objectives:**

To investigate the relationship between the eccentric calcification of aortic valve and transcatheter heart valve (THV) distortion and the impact of THV distortion on echo parameters and clinical outcomes.

**Background:**

The effects of eccentric calcification of the aortic valve on the THV distortion and the relationship between THV distortion and clinical impact were not fully understood.

**Methods:**

Patients with symptomatic severe aortic stenosis who were undergoing THV implantation were enrolled. Patients underwent preprocedural, postprocedural multislice computed tomography (MSCT), and follow-up transthoracic echocardiogram (TTE). *Delta calcium score* (ΔCS) is defined as the difference between the maximum and minimal calcium scores of the three cusps, while *valve distortion score* (VDS) is defined as the difference between the longest and shortest stent frame, as obtained using MSCT. Patients were divided into two groups according to ΔCS: “noneccentric calcification group” and “eccentric calcification group.”

**Results:**

A total of 118 patients were enrolled (59 patients in noneccentric and 59 in eccentric calcification groups). VDS was significantly lower in the noneccentric calcification group than in the eccentric calcification group (1.31 ± 0.82 mm vs. 1.73 ± 0.76 mm, *p*=0.004). VDS was not associated with the degree of paravalvular leak (PVL) and aortic valvular mean pressure gradient (AVPG) at 30-day and 1-year follow-up TTE and the cumulative rates of all-cause death and rehospitalization at 2-year clinical follow-up.

**Conclusions:**

Eccentric valvular calcification was associated with longitudinal THV distortion. However, THV distortion was not associated with PVL, AVPG, and adverse clinical events during midterm follow-up.

## 1. Introduction

Transcatheter aortic valve implantation (TAVI) is a well-established treatment for intermediate- to high-risk patients with severe aortic stenosis (AS) [1–3]. However, previous studies reported that high burden of aortic valve calcification is a risk factor for TAVI complications, such as annulus rupture and paravalvular leak (PVL) [4, 5]. Calcification is also associated with the following factors: higher rates of adverse events during percutaneous coronary interventions (PCI), peripheral vascular interventions, and endovascular therapy for abdominal aortic aneurysms [6–10]. Some previous small studies investigated the distortion of transcatheter heart valve (THV) and its impact on THV functions [11, 12]. However, the effects of eccentric calcification of the aortic valve on the distortion of THV and the impact of THV distortion on THV functions and clinical impact were not fully understood. This study aimed to investigate the relationship between the degree and eccentricity of aortic valve calcification and THV distortion and the impact of THV distortion on follow-up echo parameters and clinical outcomes after TAVI.

## 2. Methods

### 2.1. Study Population and Procedures

Symptomatic patients diagnosed with severe AS who were undergoing TAVI were enrolled in this study. AS severity was defined according to the Valve Academic Research Consortium-2 criteria [13]. All TAVI procedures were performed using the third-generation balloon expandable Edwards SAPIEN3 heart valve system (ES3, Edwards Lifesciences, Irvine, California) with fluoroscopic and echo guidance (transthoracic or transesophageal). All patients underwent transfemoral placement of the THVs, sized 20, 23, 26, and 29 mm. THV size was selected using multislice computed tomography (MSCT) [14]. Heparin was administered to all patients during the procedure. Moreover, antiplatelet therapy (aspirin or clopidogrel or both) or anticoagulant therapy was administered before and after the procedure.

### 2.2. Computed Tomography Analysis and Definition

All patients underwent pre- and post-TAVI MSCT using the SOMATOM Definition AS + CT system with 64 detectors (SIEMENS Healthcare, Erlangen, Germany). The MSCTs performed before TAVI were contrast-enhanced. Scans were performed using electrocardiography synchronized data during acquisition with a systolic phase requirement. Aortic valve calcium scores were measured for each leaflet using the pre-TAVI MSCT image. To calculate aortic valve calcium scores, calcium volume score method was used [15, 16]. We applied the calcium volume score (not the Agatston score) because calcium volume score does not rely on lesion density, and instead seeking aims to estimate the true calcium volume. Thus, we believed the calcium volume score was more appropriate for our study. *Delta calcium score* (ΔCS) is defined as the difference between the maximum and minimal three-cusp calcium scores. Patients were divided into two groups according to the ΔCS: those with lower-than-median ΔCS were assigned to the “noneccentric calcification group,” whereas those with a higher-than-median ΔCS were assigned to the “eccentric calcification group” (median ΔCS = 148.5 mm^3^).

**Figure 1:**
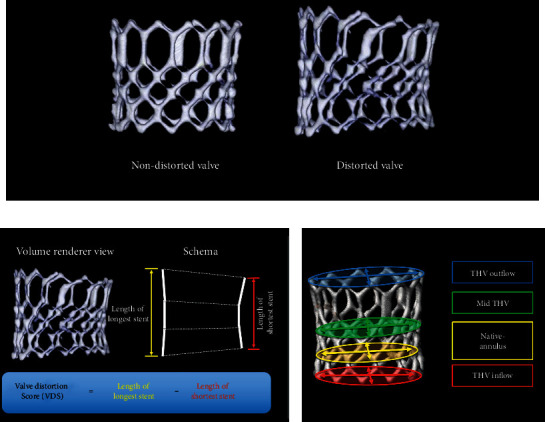
Transcatheter heart valve distortion and multislice computed tomography analysis. A normal expanded transcatheter heart valve (THV) and distorted THV in a multislice computed tomography (MSCT) volume-rendered view (a). Using the postprocedural MSCT, a 3D-volume-rendered view of THV was constituted. The length of the stent frame on the center of each cusp was measured. Valve distortion score (VDS) was defined as the difference between the longest and shortest stent frame lengths (b). The THV stent frame was geometrically evaluated for maximal and minimal orthogonal diameters at the four levels of THV: THV inflow, native annulus, mid-THV, and THV outflow. The cross-sectional eccentricity index was defined as follows: (1 − short diameter/long diameter) × 100 (c).

The MSCTs of post-TAVI procedures were usually performed before discharge and without use of contrast medium. Post-TAVI MSCT was used to create a 3D-volume-rendered view of the THV. The stent frame length was measured at each cusp. *Valve distortion score* (VDS) refers to the difference between the longest and shortest stent frame lengths (Figures 1(a) and 1(b)). The nominal ES3 stent frame lengths are 15.5 mm (20 mm THV), 18 mm (23 mm THV), 20 mm (26 mm THV), and 22.5 mm (29 mm THV). All THV stent frames were geometrically measured for the maximal and minimal orthogonal diameters at the four levels of THV: (a) THV inflow, (b) native annulus, (c) mid-THV, and (d) THV outflow [17]. Cross-sectional eccentricity index (THV) was defined as follows: (1 − short diameter/long diameter) × 100 (Figure 1(c)) [17, 18]. Valve oversizing ratio was calculated as follows: (observed THV external area at the native annulus level/native annular area − 1) × 100 [17]. All imaging constitutions and measurements were performed using a dedicated software (3mensio software; Pie Medical Imaging, Maastricht, Netherlands).

### 2.3. Study Objectives

This study aimed to evaluate (1) the relationship between eccentric aortic valve calcification and THV distortion using 3D-MSCT imaging, (2) the relationship between THV distortion and THV functions such as PVL and aortic valvular mean pressure gradient (AVPG) using follow-up transthoracic echocardiogram (TTE), and (3) the impact of THV distortion on midterm (2-year) clinical outcomes.

### 2.4. Statistical Analysis

Categorical data are presented as counts and percentages and are compared using the chi-square test or Fisher exact tests. All continuous variables were tested for normality using the Kolmogorov-Smirnov test. Continuous variables are presented as mean ± SD or median (interquartile range) for normally and nonnormally distributed variables. Between-group differences in continuous variables were compared using Student's *t*-test or Mann-Whitney *U* test. All data were analyzed using SPSS (version 25 for Windows; SPSS, Inc., Chicago Illinois) and R (version 3.0.2; The R Foundation for Statistical Computing Vienna, Austria).

## 3. Results

### 3.1. Baseline Characteristics and Clinical Outcomes

Between June 2016 and December 2018, 241 patients underwent TAVI. Among them, 139 patients underwent SAPIEN3 implantation. Of these, 19 patients who did not undergo postprocedural MSCT and two patients who died before discharge (1 patient died from acute respiratory distress syndrome and one patient died from colitis) were excluded in this study. In total, 118 patients were included. Patients' baseline data are shown in Table 1. This study included 42 males (35.6%) with mean age of 83.6 ± 5.1 years. The mean STS and Logistic Euro scores were 8.2 ± 7.5 and 21.0 ± 12.9, respectively. The prevalence of chronic obstructive pulmonary disease was significantly higher in the eccentric calcification group, whereas the rate of prior PCI was higher in the noneccentric calcification group. The prevalence of diabetes mellitus was shown to be higher in the eccentric calcification group.

### 3.2. MSCT Parameters

Preprocedural MSCT parameters are shown in Table 2. Among the three cusps in the 118 patients, the highest calcium score was observed in the noncoronary cusps (255.4 ± 159.8 mm^3^).

Of these, 59 (50%) were assigned to the noneccentric calcification group (ΔCS < 148.5 mm^3^), while 59 patients (50%) were assigned to the eccentric calcification group (ΔCS ≥ 148.5 mm^3^). The total calcium score of all the cusps was significantly lower in the noneccentric calcification group compared with those of the eccentric group (429.6 ± 333.3 mm^3^ vs. 733.9 ± 335.8 mm^3^, *p* < 0.001). Furthermore, the noncoronary cusp and the right-coronary cusp calcium scores were also significantly lower in the noneccentric calcification group compared with those of the eccentric calcification group.

**Figure 2:**
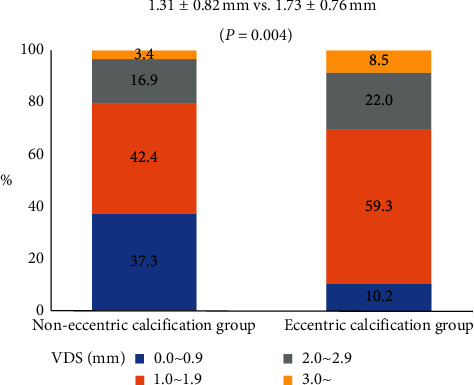
The effects of aortic valvular calcium eccentricity on the valve distortion score. Valve distortion score (VDS) was significantly lower for the noneccentric calcification group compared with those of the eccentric calcification group.

On average, patients underwent MSCT 5.7 days after TAVI. Table 2 shows the parameters of THV measured using postprocedural MSCT. VDS was significantly lower for the noneccentric calcification group than those in the eccentric calcification group (1.31 ± 0.82 mm vs. 1.73 ± 0.76 mm, *p* < 0.004) (Figure 2). The longest stent frames were most frequently observed in the noncoronary cusp, while the shortest stent frames were most often observed in the left-coronary cusp. No significant between-group differences were observed in cross-sectional eccentricity indexes at any part of the THV levels (THV inflow, native annulus, mid-THV, and THV outflow).

### 3.3. Procedural Details

Procedural details are presented in Table 3. No significant difference was observed in the THV size between noneccentric and eccentric calcification groups. One patient needed a second valve due to valve migration. The most frequently used THVs were 23 mm in both groups. Predilatation and postdilatation were performed in 63 (53.4%) and 31 (26.3%) patients, respectively. The rate of predilatation was lower in the noneccentric group, compared with those of the eccentric calcification group. Nominal delivery balloon volume, overfilling volume (≥1 mL additional volume), and underfilling volume (≥1 mL less volume) were used in 63 (53.4%), two (1.7%), and 53 (44.9%) patients, respectively. No significant difference was observed in the delivery balloon volume between the two groups.

### 3.4. TTE Parameters

TTE parameters are shown in Table 4. No significant difference was observed in the aortic valve area (AVA) and AVPG between noneccentric and eccentric calcification groups using the preprocedural TTE (AVA; 0.66 ± 0.15 cm^2^ vs. 0.64 ± 0.14 cm^2^, *p*=0.458, AVPG; 46.6 ± 20.2 mmHg vs. 49.7 ± 17.2 mmHg, *p*=0.376). The noneccentric calcification group had a less maximum aortic gradient than the eccentric calcification group (75.4 ± 26.0 mmHg vs. 87.1 ± 26.9 mmHg, *p*=0.018).

**Figure 3:**
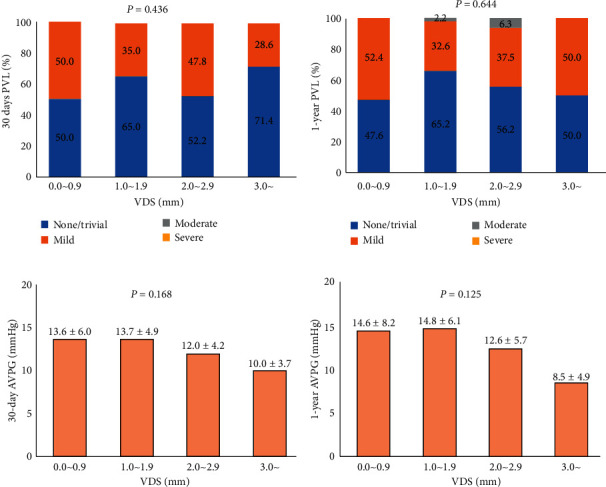
The association of valve distortion score with paravalvular leak and aortic valvular pressure gradient. The degree of valve distortion score (VDS) was not associated with a 30-day paravalvular leak (PVL) (a) and aortic valvular pressure gradient (AVPG) (c). The VDS was also not associated with the degree of PVL (b) and AVPG (d) at 1-year follow-up transthoracic echocardiogram.

No significant difference was observed in the degree of PVL and AVPG between noneccentric calcification group and eccentric calcification group at 30-day follow-up TTE (Table 4). In addition, VDS was not associated with the degree of PVL and AVPG (Figures 3(a) and 3(c)).

One-year follow-up TTE data were obtained in 89 (75.4%) patients. No significant difference was observed in the degree of PVL and AVPG between noneccentric calcification group and eccentric calcification group, and VDS was not associated with the degree of PVL and AVPG at 1-year follow-up TTE (Table 4 and Figures 3(b) and 3(d)).

### 3.5. Clinical Outcomes

**Figure 4:**
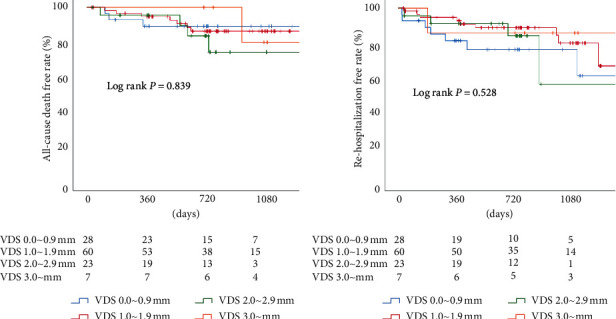
Kaplan-Meier curves of all-cause death free and rehospitalization free survival rates. There were no significant differences in the all-cause death free and rehospitalization free survival rates among various valve distortion scores (VDS).

Median clinical follow-up period was 743 (interquartile range: 504–1,074) days. No adverse events were observed including all-cause death and requiring hospitalization for valve-related symptoms or worsening congestive heart failure up to 30 days of follow-up. The cumulative rates of all-cause death (VDS 0.0∼0.9 mm group vs. VDS 1.0∼1.9 mm group vs. VDS 2.0∼2.9 mm group vs. VDS 3.0∼ mm group: 10.7% vs. 11.7% vs. 8.7% vs. 0.0%; *p*=0.800), rehospitalization for valve-related symptoms or worsening congestive heart failure (21.4% vs. 10.0% vs. 13.0% vs. 14.3%; *p*=0.545), and valve thrombosis (7.1% vs. 5.0% vs. 4.3% vs. 0.0%; *p*=0.885) at 2 years were not significantly different by degree of VDS.  Figure 4 shows Kaplan-Meier curves of all-cause death free and rehospitalization free survival rates.

## 4. Discussion

**Figure 5:**
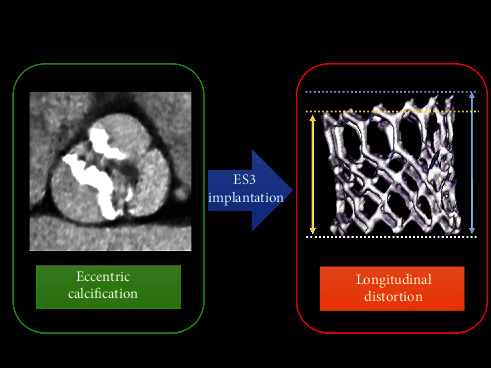
Eccentricity of aortic valvular calcification and transcatheter heart valve distortion. Eccentricity of aortic valvular calcification was associated with longitudinal distortion of Edwards SAPIEN3 (ES3, Edwards Lifesciences, Irvine, California).

Findings showed that (1) eccentric calcification was associated with greater longitudinal THV distortion than noneccentric calcification of the aortic valve (Figure 5). On the other hand, (2) eccentric calcification was not associated with the cross-sectional THV eccentricity. In addition, (3) longitudinal THV distortion was not associated with PVL grade and AVPG at 30-day and 1-year TTE. Moreover, (4) longitudinal THV distortion did not affect the cumulative rates of adverse clinical events (all-cause death, rehospitalization, and valve thrombosis) at 2-year clinical follow-up.

### 4.1. Eccentric Calcification and THV Distortion

Studies have revealed that eccentric calcification caused device deformation and adverse events in procedures other than TAVI. During PCI procedures, stent implantation in severely calcified lesions can result in less optimal stent expansion, mispositioning, and stent fracture [6–8, 19]. In addition, calcified plaques are associated with side branch stenosis after main branch stenting due to stent migration to the side opposite to the side where there is severe calcification [20]. Eccentric calcification is also associated with increased risk of endoleak in the procedure of endovascular therapies for abdominal aortic aneurysms and hemodynamic instability in the procedure of carotid angioplasty [21, 22]. Thus, we evaluated the effects of eccentric calcification of the aortic valve on the distortion of THV and the impact of THV distortion on THV functions and clinical impact in current study. Some potential mechanisms of these THV distortions were considered in this study. Severe calcification sites push the THV toward the mild calcification sites. Furthermore, because the THV delivery balloon is semicompliant, the delivery balloon may expand in a nonuniform manner within the eccentric calcification site. The cells of the THV stent frame are fully opened in mildly calcified sites, but not in sites with severe calcification. Thus, longitudinal THV distortions can possibly occur in aortic valves with eccentric calcification.

No significant between-group difference was observed in the cross-sectional eccentricity index between the noneccentric and eccentric calcification groups. This might be due to improvements in radial strength as devices used evolved (ES3 has larger cells and wider strut angles than the SAPIEN XT; Edwards Lifesciences, Irvine, California) [23].

### 4.2. Impacts of THV Distortion on TTE Parameters and Clinical Outcomes

In spite of these adverse effects related to device deformation due to severe eccentric calcification, THV distortion did not affect the severity of PVL and AVPG, as measured during the 30-day follow-up TTE in this study. This significant PVL (>moderate) low rate may be due to the improvements made to the THV device. The S3 has an outer polyethylene terephthalate cuff, which prevents significant PVL [23].

Additionally, the THV distortion was not associated with TTE parameters (degree of PVL and AVPG) at 1-year follow-up. Previous studies reported that THV has good durability in mid- to long-term follow-ups [24–27]. However, the association between THV distortion due to eccentric calcification and THV durability is not well known. This study revealed that THV maintains excellent functions regardless of longitudinal THV distortion in midterm follow-up TTE.

Moreover, this study revealed that the degree of THV distortion was not associated with the following adverse events: cumulative rates of all-cause death, rehospitalization for valve-related symptoms or worsening congestive heart failure, and valve thrombosis at 2-year clinical follow-up. The fact that THV kept good functions regardless of THV distortion might contribute to these positive clinical outcomes.

There are some reports which evaluated the durability of THV [28]. Most of them showed the favorable durability of THV. However, the relationship between THV function (durability) and severe eccentric calcification or THV distortion has not been fully elucidated. In addition, Blackman et al. reported that median duration of moderate structural valve degeneration was about 6 years following TAVI [24]. Thus, we cannot easily conclude that THV distortion does not affect the THV function, although current study added the novel information that severe eccentric calcification was associated with longitudinal THV distortion, but longitudinal THV distortion was not associated with THV dysfunction and adverse clinical events during midterm follow-up. Studies with large number of patients and long-term follow-up are warranted.

### 4.3. Study Limitations

This study has several limitations. First, this was an observational single-center study. Second, this study was conducted with a relatively small patient cohort. Third, patients without postprocedure MSCT scans were excluded in this study. Therefore, selection bias cannot be excluded. Fourth, this study only evaluated the relationship between the eccentricity of calcification in aortic valvular leaflet and THV distortion. Thus, we cannot exclude the possibility that calcium outside the valve (such as left ventricular outflow tract) affected the THV distortion. Fifth, we focused on the relationship between degree of VDS and PVL in the current study. However, there is a possibility that other confounding factors might affect the degree of PVL. Sixth, the number of patients with VDS larger than 2 mm was relatively small (25.4%). Thus, we cannot exclude the possibility that the results were affected by the small number of them. To solve this problem, further multicenter registry with large number of patients is needed. Finally, this study was conducted with only midterm follow-up. Therefore, whether this distortion of THV impacts clinical outcomes or not is unknown within the context of long-term follow-up. Large numbers of patients and longer follow-up studies are needed. Several prospective randomized trials (the PARTNER 3 trial, the Medtronic Evolut low-risk trial) showed excellent short-term outcomes after TAVI in low-risk patients [29, 30]. Based on these trials, indication of TAVI is expected to expand. Before that, long-term results need to be evaluated whether THV distortions are associated with deterioration or adverse clinical outcomes.

## 5. Conclusions

Eccentricity of aortic valvular calcification was associated with longitudinal, but not cross-sectional, distortion of the THV stent frame. However, this longitudinal THV distortion was not associated with the degree of PVL and AVPG, and clinical adverse events in midterm follow-up.

## Figures and Tables

**Table 1:** Patients characteristics.

	Overall (*n* = 118)	Noneccentric calcification group (*n* = 59)	Eccentric calcification group (*n* = 59)	*p* value
Age (y)	83.6 ± 5.1	83.0 ± 4.6	84.1 ± 5.5	0.241
Male, *n* (%)	42 (35.6)	17 (28.8)	25 (42.4)	0.124
Body mass index (kg/m^2^)	22.7 ± 4.1	23.2 ± 4.6	22.2 ± 3.5	0.201
STS score for mortality	8.2 ± 7.5	7.3 ± 4.0	9.2 ± 9.7	0.177
Logistic euroscore	21.0 ± 12.9	19.2 ± 10.7	22.8 ± 14.7	0.123
Hypertension, *n* (%)	84 (71.2)	45 (76.3)	39 (66.1)	0.223
Dyslipidemia, *n* (%)	47 (39.8)	21 (35.6)	26 (44.1)	0.347
Diabetes mellitus, *n* (%)	29 (24.6)	15 (25.4)	25 (42.4)	0.052
Chronic kidney disease, *n* (%)	67 (56.8)	30 (50.8)	37 (62.7)	0.193
Cerebral vascular disease, *n* (%)	44 (37.3)	20 (33.9)	24 (40.7)	0.446
Peripheral vascular disease, *n* (%)	37 (31.4)	16 (27.1)	21 (35.6)	0.427
Chronic obstructive pulmonary disease, *n* (%)	24 (20.3)	7 (11.9)	17 (28.8)	0.022
Atrial fibrillation, *n* (%)	41 (34.7)	17 (28.8)	24 (40.7)	0.176
Prior permanent pacemaker, *n* (%)	4 (3.4)	2 (3.4)	2 (3.4)	1.000
Prior myocardial infarction, *n* (%)	10 (8.5)	4 (6.8)	6 (10.2)	0.509
Prior PCI, *n* (%)	24 (20.3)	17 (28.8)	7 (11.9)	0.022
Prior CABG, *n* (%)	5 (4.2)	1 (1.7)	4 (6.8)	0.364
Prior BAV, *n* (%)	2 (1.7)	1 (1.7)	1 (1.7)	1.000

Values are expressed as either mean ± SD or absolute frequency (percentage). STS, Society of Thoracic Surgeons. EuroScore, European System for Cardiac Operative Risk Evaluation. PCI, percutaneous coronary intervention. CABG, coronary artery bypass grafting. BAV, balloon aortic valvuloplasty.

**Table 2:** Multislice computed tomography results.

	Overall (*n* = 118)	Noneccentric calcification group (*n* = 59)	Eccentric calcification group (*n* = 59)	*p* value
*Preprocedural MSCT parameters*				
Calcium score				
All cusps (mm^3^)	581.8 ± 366.5	429.6 ± 333.3	733.9 ± 335.8	<0.001
Noncoronary cusp (mm^3^)	255.4 ± 159.8	158.0 ± 119.5	352.8 ± 133.9	<0.001
Right-coronary cusp (mm^3^)	184.4 ± 168.0	140.2 ± 121.7	228.5 ± 195.3	0.004
Left-coronary cusp (mm^3^)	142.7 ± 105.3	133.1 ± 107.8	152.3 ± 102.7	0.326
Delta calcium scores (ΔCS) (mm^3^)	175.8 ± 135.3	80.4 ± 37.0	271.3 ± 130.6	<0.001

*Postprocedural MSCT parameters*				
THV stent frame				
Length of the longest stent frame (mm)	20.81 ± 2.04	20.33 ± 2.02	21.29 ± 1.95	0.010
Length of the shortest stent frame (mm)	19.29 ± 1.82	19.03 ± 1.81	19.56 ± 1.80	0.115
Valve distortion score (VDS) (mm)	1.52 ± 0.82	1.31 ± 0.82	1.73 ± 0.76	0.004
THV cross-sectional eccentricity index				
THV inflow	4.08 ± 2.40	4.04 ± 2.47	4.13 ± 2.35	0.841
THV annulus	4.49 ± 2.83	4.48 ± 2.86	4.49 ± 2.83	0.983
THV mid	4.23 ± 2.71	3.90 ± 2.23	4.56 ± 3.11	0.189
THV outflow	2.48 ± 1.50	2.24 ± 1.49	2.73 ± 1.48	0.080

Values are expressed as either mean ± SD. THV, transcatheter heart valve. MSCT, multislice computed tomography.

**Table 3:** Procedural details.

	Overall (*n* = 118)	Noneccentric calcification group (*n* = 59)	Eccentric calcification group (*n* = 59)	*p* value
THV size (mm)				0.400
20	13 (11.0)	7 (11.9)	6 (10.2)	
23	60 (50.8)	34 (57.6)	26 (44.1)	
26	40 (33.9)	16 (27.1)	24 (40.7)	
29	5 (4.2)	2 (3.4)	3 (5.1)	
Access route				
Transfemoral, *n* (%)	118 (100)	59 (100)	59 (100)	1.000
Predilatation, *n* (%)	63 (53.4)	23 (39.0)	40 (67.8)	0.003
Postdilatation, *n* (%)	31 (26.3)	18 (30.5)	13 (22.0)	0.403
Delivery balloon volume				0.062
Nominal volume, *n* (%)	63 (53.4)	36 (61.0)	27 (45.8)	
Over filling volume, *n* (%)	2 (1.7)	2 (3.4)	0 (0)	
Under filling volume, *n* (%)	53 (44.9)	21 (35.6)	32 (54.2)	
Valve oversizing ratio (%)	21.1 (12.3–26.6)	19.9 (10.4–25.7)	21.2 (14.0–28.1)	0.176

Values are expressed as median (interquartile) or absolute frequency (percentage). THV, transcatheter heart valve.

**Table 4:** Transthoracic echocardiogram parameters.

	Overall	Noneccentric calcification group	Eccentric calcification group	*p* value
*Peri-procedural TTE parameters*	*n* = 118	*n* = 59	*n* = 59	
Preprocedural TTE				
Aortic valve area (cm^2^)	0.65 ± 0.14	0.66 ± 0.15	0.64 ± 0.14	0.458
Mean gradient (mmHg)	48.2 ± 18.8	46.6 ± 20.2	49.7 ± 17.2	0.376
Maximum gradient (mmHg)	81.2 ± 27.0	75.4 ± 26.0	87.1 ± 26.9	0.018
Left ventricular ejection fraction (%)	59.8 ± 11.7	59.4 ± 12.7	60.3 ± 10.6	0.663
Postprocedural TTE				
Aortic valve area (cm^2^)	1.72 ± 0.30	1.67 ± 0.26	1.78 ± 0.33	0.043
Mean gradient (mmHg)	11.5 ± 4.6	11.8 ± 4.5	11.1 ± 4.8	0.424
Maximum gradient (mmHg)	22.3 ± 8.3	22.8 ± 8.4	21.7 ± 8.3	0.485
Paravalvular leak, *n* (%)				0.647
None/trivial	94 (79.7)	48 (81.4)	46 (78.0)	
Mild	24 (20.3)	11 (18.6)	13 (22.0)	
Moderate	0 (0)	0 (0)	0 (0)	
Severe	0 (0)	0 (0)	0 (0)	
30-day TTE				
Aortic valve area (cm^2^)	1.66 ± 0.31	1.62 ± 0.27	1.71 ± 0.34	0.117
Mean gradient (mmHg)	13.1 ± 5.0	14.0 ± 5.0	12.3 ± 5.0	0.079
Maximum gradient (mmHg)	25.0 ± 9.7	26.1 ± 9.4	23.8 ± 9.9	0.194
Paravalvular leak, *n* (%)				0.261
None/trivial	70 (59.3)	38 (64.4)	32 (54.2)	
Mild	48 (40.7)	21 (35.6)	27 (45.8)	
Moderate	0 (0)	0 (0)	0 (0)	
Severe	0 (0)	0 (0)	0 (0)	

*1-year TTE*	*n* = 89	*n* = 40	*n* = 49	
Aortic valve area (cm^2^)	1.59 ± 0.34	1.56 ± 0.32	1.62 ± 0.36	0.394
Mean gradient (mmHg)	13.9 ± 6.6	14.0 ± 6.4	13.9 ± 6.8	0.901
Maximum gradient (mmHg)	26.3 ± 12.1	25.7 ± 12.1	26.7 ± 12.2	0.703
Paravalvular leak, *n* (%)				0.172
None/trivial	52 (58.4)	27 (67.5)	25 (51.0)	
Mild	35 (39.3)	13 (32.5)	22 (44.9)	
Moderate	2 (2.2)	0 (0.0)	2 (4.1)	
Severe	0 (0.0)	0 (0.0)	0.0 (0.0)	

Values are expressed as either mean ± SD or absolute frequency (percentage). TTE, transthoracic echocardiogram.

## Data Availability

The data used to support the findings of this study are included within the article.

## Abbreviations

AS: Aortic stenosis
AVA: Aortic valve area
AVPG: Aortic valvular mean pressure gradient
ΔCS: Delta calcium score
ES3: Edwards SAPIEN3
MSCT: Multislice computed tomography
PCI: Percutaneous coronary interventions
PVL: Paravalvular leak
TAVI: Transcatheter aortic valve implantation
THV: Transcatheter heart valve
TTE: Transthoracic echocardiogram
VDS: Valve distortion score.
